# Wireless power system for left ventricular assist device: Influence of coil design and tissue behavior on efficiency

**DOI:** 10.2478/joeb-2025-0010

**Published:** 2025-06-16

**Authors:** Hawraa A. Almorshidi, Ali Jafer Mahdi, Manal Hussain Nawir

**Affiliations:** Department of Electrical and Electronic Engineering, University of Kerbala, 56001 Karbala, Iraq; College of Information Technology Engineering, Al-Zahraa University for Women, 56001, Karbala, Iraq

**Keywords:** Left Ventricular Assist Device, Wireless Power Transfer system, Litz wire, Mutual inductance, Multilayer coil, Buck-boost converter, Human tissues

## Abstract

This paper proposes a compact wireless power transfer (WPT) system designed to energize an implanted heart pump. The design integrates several power converters: a buck-boost converter supplied by a 14-volt battery, an H-bridge inverter, a low-pass filter, and a resonant inductive coupling WPT unit. A resistive load of 40 ohms is used to simulate the equivalent pump's operation. To improve efficiency and limit power losses caused by high-frequency skin effects, a Litz wire is utilized. Consequently, a multi-layer transmission coil structure is employed to strengthen coupling and ensure deeper field penetration. The system operates in an open-loop configuration with manual adjustment of the DC-DC converter's duty cycle. A frequency of 6.78 MHz is selected based on the Industrial, Scientific, and Medical band due to its recognized safety and its ability to achieve deeper penetration into biological tissues. To optimize the design, precise mathematical modeling of both the WPT system and the tissue layers is conducted, simulating their impact on electromagnetic field behavior. Simulation results demonstrate an impressive power transfer efficiency of 91% across a separation of 60 mm. It is worth noting that most existing studies focus on low-power wireless energy delivery for internal medical devices; this research advances the field by targeting higher power demands, positioning it as a practical solution for critical applications like heart assist pumps.

## Introduction

On a global scale, advanced heart failure is expected to increase dramatically in the upcoming years. In the United States, 8 million people are predicted to have heart failure by 2030 [1]. The Eurotransplant Annual Report 2016 [2] indicates a significant increase in patients on the waiting list for heart transplants. This growing demand has prompted the development of Implantable Mechanical Cardiac Devices (IMCDs) such as Total Artificial Hearts (TAHs) and Left Ventricular Assist Devices (LVADs). The TAHs are designed to replace the human heart entirely, while the LVADs are designed to support a weakened heart, and they are commonly used as a long-term treatment [3,4]. The LVADs consist of a hydraulic pump implanted within the body, receiving electrical energy from external batteries via a driveline. Despite its clinical importance, these devices still face critical limitations—foremost among them are driveline infections, which remain the most significant and life-threatening complication associated with long-term LVAD use [5]. This challenge makes Wireless Power Transfer (WPT) technology a promising engineering solution to eliminate physical connections and reduce infection risks. While WPT has been extensively investigated in Implantable Medical Devices (IMDs), most reported studies focus on low-power, shallow implants such as subcutaneous sensors, retinal implants, and smart contact lenses. For instance, [6] presented a wireless power transmission system for a smart contact lens using magnetic resonance inductive coupling at 13.56 MHz and 350 mW, achieving a transmission efficiency of 17.5% over 20 mm. In [7], a technique based on capacitive coupling called Capacitively Coupled Conductive Trans-cutaneous Energy Transfer (CCCTET) was employed to deliver 10 mW at 6.78 MHz, with an efficiency of just 0.45%.

By contrast, research efforts addressing high-power implantable devices such as LVADs using WPT are extremely limited. One notable example is [8], which proposed a resonant inductive coupling system for LVADs and achieved a power transfer efficiency of around 85–86%. However, the previous study relied on large elliptical coils wrapped around the torso and used ferrite layers in the implanted unit to improve coupling, which increased the system's size and complexity. It also employed conventional wire with higher resistance, leading to resistive losses and potential tissue heating. These limited studies indicate a clear gap in the literature concerning high-efficiency and safe wireless power transfer systems for high-power, deeply implanted medical devices.

**Fig. 1:**
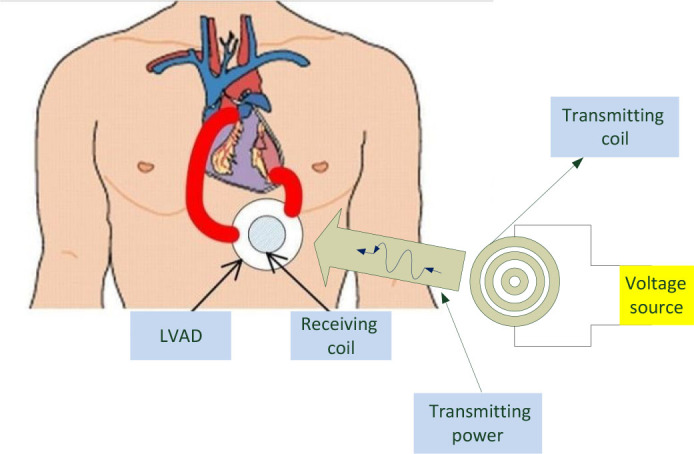
Wireless power transfer of LVAD.

In response to this need, the present study aims to develop and enhance a wireless power transfer (WPT) system based on resonant inductive coupling to power the LVAD, as illustrated in Fig. 1. The proposed design employs a compact spiral coil placed on the abdomen without ferrite layers, utilizes low-loss Litz wire, and incorporates accurate tissue modeling. The system targets optimal power transfer efficiency for separation distances ranging from 20 mm to 80 mm. This research also investigates the influence of human tissue on magnetic coupling between coils and analyzes the effects of electromagnetic waves on tissue displacement and dehydration. A mathematical model is developed to assess the design's feasibility and simulate its biological effects. The system was implemented and evaluated using MATLAB/Simulink, paving the way for future experimental validation and clinical application.

## Materials and methods

**Fig 2.:**
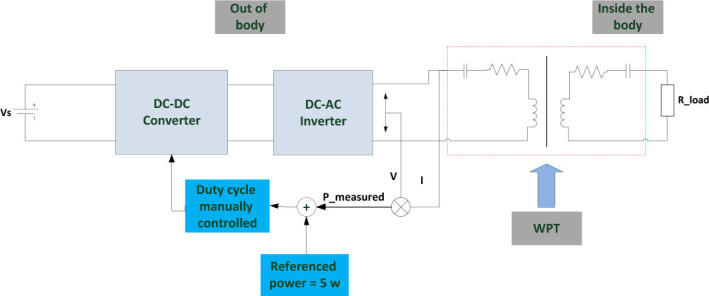
Block diagram of the WPT system.

Fig. 2 shows the block diagram of the proposed WPT system, which comprises a 14V battery, a buck-boost converter, and an inverter. As explained in the later sections, Litz wire and multilayer coil technologies were employed in the transmission to enhance its efficiency and efficacy.

### LVAD characteristics

A brushless DC motor drive is utilized in the LVAD, which is implanted within the human body [8]. External batteries always power the LVAD. The batteries are connected to the implanted device via a wire that passes through the abdomen. The LVAD is categorized as a high-power implanted device, necessitating an average output of approximately 5 watts and a peak power of around 20 watts under all operational conditions [8]. The load is represented as a resistance of around 40 ohms by Eq. (1) when the voltage is 14 V.

(1) $$R=\frac{V^{2}}{P}$$

### Wireless Power Transfer System (WPTS)

Wireless transmission systems are classified into two main categories: far-field and near-field. Far-field power transmission includes microwave and ultrasound technologies, both of which operate at radio frequencies (RF) (300 MHz – 300 GHz) [9]. Near-field transmission includes inductive coupling and capacitive coupling technologies. In capacitive coupling, power is transmitted by an electric field, while in inductive coupling, power is transmitted by a magnetic field. Generally, the frequencies in this category are lower than the far-field frequencies, usually above 10 kHz to a few MHz [10]. Near-field transmission systems are superior for medical applications due to their minimal coil losses and tolerance to tissue attenuation. This study employs an inductive coupling system for wireless power transfer because of its superior flexibility in coil distance and alignment compared to capacitive coupling, which necessitates a restricted distance and precise plate alignment [11].

### Inductive coupling

**Fig. 3:**
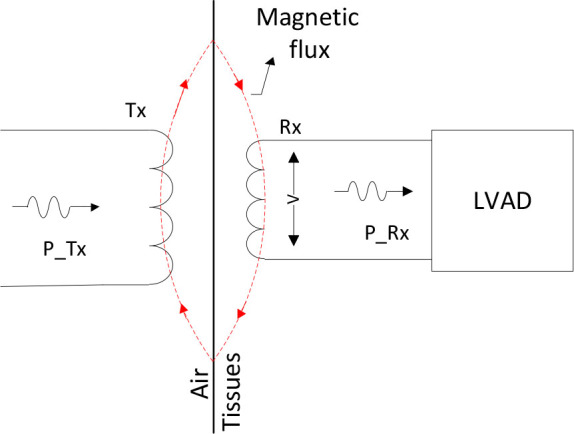
Block diagram of the proposed system.

This system employs two distinct coils, designated as Tx and Rx. The Tx coil is the transmitting coil, connected to the voltage source and positioned at the output to the human body, forming the transmitting circuit. The Rx coil is the secondary or receiving coil, embedded within the human body and connected to the pump, thereby forming the receiving circuit, as illustrated in Fig. 3.

**Fig. 4:**
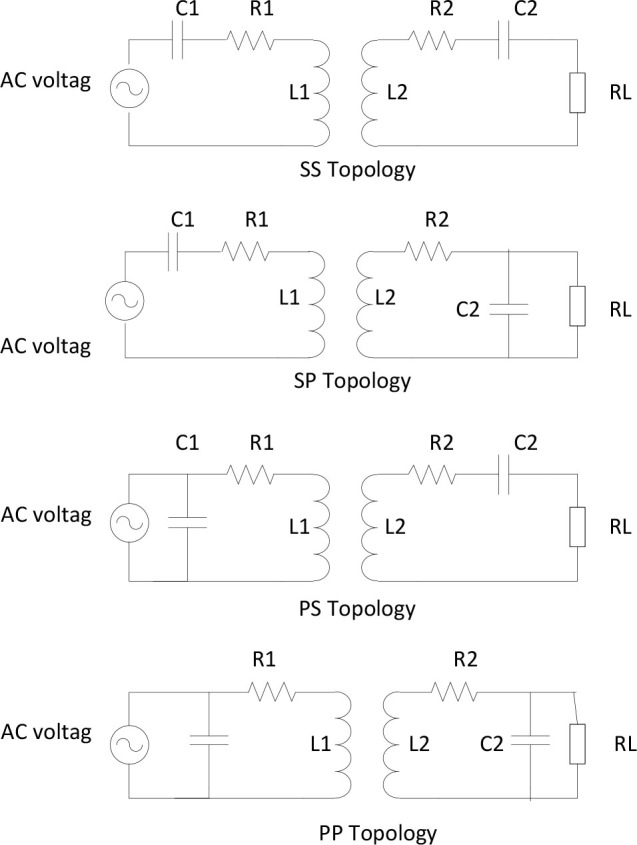
Resonant inductive coupling.

To enhance power transfer efficiency, resonant inductive coupling is employed instead of conventional inductive coupling. This is because it can transmit greater power between the coils over extended distances [11]. The rationale is that operating at resonance minimizes the system's impedance, reducing losses. This provides an additional significant benefit, as the heat produced in the receiving circuit will decrease with reduced losses. Another benefit of resonance is its ability to reduce interference with other waves present in the medium. Resonance is attained by compensating the inductive reactance of the transmitting and receiving coils using compensating capacitors. As shown in Fig. 4, the system can incorporate capacitors in several configurations, specifically Series-Series (SS), Series-Parallel (SP), Parallel-Series (PS), and Parallel-Parallel (PP).

### Selection of transmission frequency

When tissues are exposed to high-frequency electro-magnetic waves, such as microwaves, polar molecules like water (H_2_O) oscillate in response to the wave's electric field. The oscillations induce friction among the molecules, leading to heat generation that escalates with increased frequency, known as dielectric heating [24]. This process results in water evaporation from the tissues, leading to desiccation, particularly of muscle tissue [29]. The selection of operating frequency is essential as it directly correlates with the system's safety and efficiency. In [12], the optimal frequency for deep implantation is 4 MHz to obtain the highest electromotive force for a small coil inside the human body within safe limits. This frequency is not designated as unlicensed and necessitates a special license, as it lies outside the Industrial, Scientific, and Medical (ISM) bands.

The ISM bands are scientifically designated frequencies that can be utilized without specific licenses; examples include 6.78 MHz, 13.56 MHz, and 2.4 GHz [13]. The frequency of 6.78 MHz was chosen as it is the closest ISM frequency to 4 MHz. Furthermore, the chosen frequency of 6.78 MHz resides within the ISM bands, which is internationally acknowledged and controlled for non-communication purposes, encompassing medical devices. It has been widely adopted in various wireless power transfer applications due to its balance between efficiency and safety, and it complies with international standards such as FCC (Part 18) and IEC 60601-1 for electromagnetic compatibility in medical environments [13].

Skin and proximity effects in coils are induced by high frequencies, such as 6.78 MHz, resulting in an increase in their equivalent resistance [14]. This resistance immediately decreases efficiency by increasing coil heat and losses. In the receiving coil, this is particularly inappropriate, as it has the potential to cause tissue damage. A Litz wire is employed to mitigate proximity and the skin effects [14,15].

### Coil design

The design of the coil is essential for high-performance WPT. Therefore, a universal coil design technique must be developed, especially when using a miniature Rx coil. This study recommends designing WPT systems with significantly larger Tx coils and tiny Rx coils. A Litz wire was used to design these coils to mitigate skin and proximity effects [14,15]. Forming a solid coil consisting of individually insulated strands ensures equal current distribution. Designing a Litz wire is necessary to ascertain the number of strands and compute the coil's equivalent resistance. [15] presents a simple method for determining the number of strands of the Litz wire and calculating the coil resistance, demonstrating equivalence to more complex methods in other studies. The first step in the design of the Litz wire is calculating the skin depth by Eq. (2) [15]:

(2) $$\delta =\sqrt{\frac{\rho }{\pi f\mu_{\circ }}}$$

where:

*ρ*: The resistivity of the conductor is equal to (1.72 × 10^−8^ Ωm) for copper at room temperature.
*f*: The frequency of a sinusoidal current in the windings.
*μ*_○_ : The permeability of free space (4*π* × 10^−7^).

Using SI units for all variables in this equation yields skin depth in meters. To guarantee that the current penetrates the entire cross-sectional area of the strand, the strand diameter (*ds*) is selected to be equal to or less than the skin depth (*ds* ≤ *δ*), hence minimizing the skin effect as much as possible. The current study utilizes the data sheet from [15] to determine the ideal characteristics of the Litz wire for an inexpensive design. In the second step, the number of strands is determined using Eq. (3) [15]:

(3) $$n_{s}=k\frac{\delta^{2}b}{N}$$

where:

k: A constant corresponding to each strand diameter as specified in the data sheet presented in [15].
b: Represents the breadth of a winding that intersects with another winding.
N: Represents the number of coil turns.

The values of *n_s_* derived from Eq. (3) should not be regarded as precise, as values 25% more or 25% less than *n_s_* may also be acceptable. The third step involves calculating the AC resistance of the coil according to Eq.(4) [15]:

(4) $$F_{r}=\frac{R_{\mathrm{ac}}}{R_{\mathrm{dc}}}=1+\frac{{\left(\pi \mathrm{nN}\right)}^{2}d_{s}{}^{6}}{192.\delta^{2}b^{2}}$$

(5) $$R_{\mathrm{dc}}=\frac{l\left(\mathrm{mm}\right)}{\sigma n\pi r_{s}{}^{2}}$$

where *Fr* represents the AC resistance factor for any number of strands, *l* is the length of the wire, *σ* is a constant, and its value is 58,000, and r is the radius of one strand. The utilized coil is a flat spiral design, optimizing the circular configuration of the LVAD to maximize spatial occupancy. The self-inductance of the coil is calculated using Eq.(6) [16]:

(6) $$L=\frac{N^{2}{\left(R_{\mathrm{out}}-N(w+s)\right)}^{2}}{16R_{\mathrm{out}}+28N(w+s)}\frac{39.37}{10^{6}}$$

where:

L: Is the self-inductance in Henry.
*d_out_* : Is the outer radius of the coil.
W: The diameter of the wire utilized for coil winding.
S: The channel width separating two adjacent wires.

**Table 1:** Parameters of the coils.

Number of turns for the primary and secondary coils (*N*_1_ & *N*_2_)	50 & 15
The outer radius of the primary and secondary coil (*R*_*O*1_ & *R*_*O*2_)	(112 & 35) mm
Inner radius of the primary and secondary coil (*R*_*i*1_ & *R*_*i*2_)	13 mm
Space between turns (*S*_1_ & *S*_2_)	(1 & 0.5) mm
Number of strands (*N*_*s*1_ & *N*_*s*2_)	(103 & 345)
Wire diameter (W)	1 mm

### Mutual inductance calculation

**Fig. 5:**
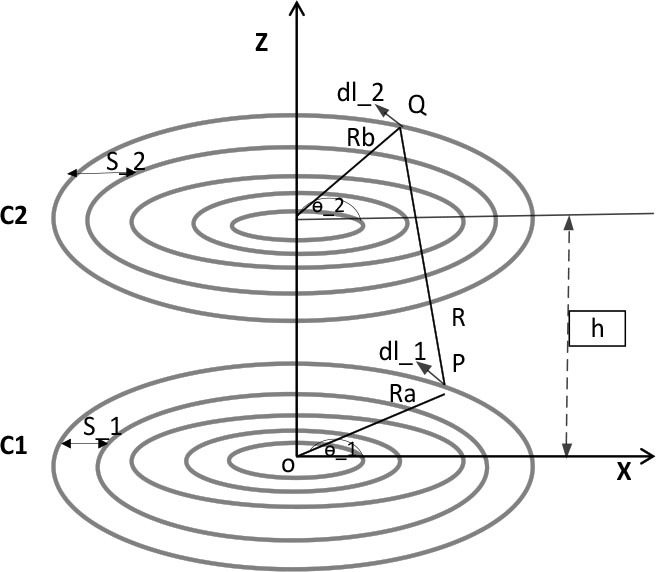
Aligned circular planar spiral coil.

Mutual inductance denotes a coil's capacity to induce an electric voltage in the other coil. The coil utilized is a planar spiral. [17] proposes a simplified method for determining M based on Neumann's integral. Two opposing coils *C*_1_, and *C*_2_, are presumed to extend along the x and z axes, with their center located at the origin, as shown in Fig. 5. The two coils are separated by h. P and Q are the tangential elements on the *C*_1_ and *C*_2_. *R_a_* and *R_b_* represents the distance from the origin point of the two coils to the tangential elements, and it is given by Eq.(7) and Eq.(8) [17]. *θ*_1_ represents the gap between them, which represents the angle of *R_a_* relative to the x-axis of *C*_1_, and *θ*_2_ is the angle of *C*_2_.

(7) $$R_{a}=R_{I1}+a\theta_{1}$$

(8) $$R_{b}=R_{I2}+a\theta_{2}$$

where a is a pitch factor and is given by the following equation:

(9) $$a=\frac{s}{2\pi }$$

The mutual inductance is determined using the Neumann equation as follows:

(10) $$M=\frac{\mu \circ }{4\pi }\oint_{C_{1}}\oint_{C_{2}}\frac{dl_{1}dl_{2}}{R}$$

where, *dl*_1_ and *dl*_2_ are line elements. In [17], the value of M is computed in two distinct scenarios: first, using Eq. (11) for optimal alignment of the transmitting and receiving coils, and second, employing Eq. (12) for a lateral misalignment by x [17]. These two equations have been adopted in this paper.

(11) $$\begin{array}{l} M=\frac{\mu_{\circ }}{4\pi }\iint_{2\pi N}\frac{j\;d\theta_{1}d\theta_{2}}{\sqrt{{\left(R_{i1}+a_{1}\theta_{1}\right)}^{2}+{\left(R_{i2}+a_{2}\theta_{2}\right)}^{2}-2j+h^{2}}}\\ j=\left(R_{i1}+a_{1}\theta_{1}\right)\left(R_{i2}+a_{2}\theta_{2}\right)\cos\left(\theta_{2}-\theta_{1}\right) \end{array}$$

**Fig. 6:**
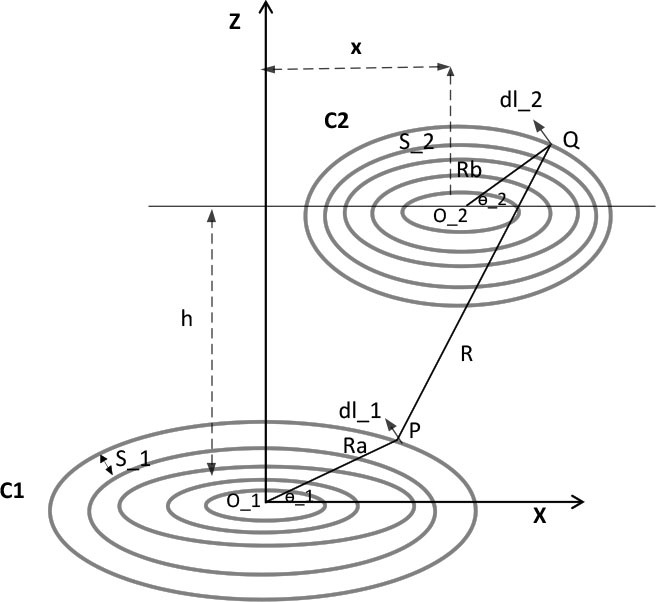
Planar spiral coil with lateral misalignment.

In magnetically coupled coils, lateral misalignment often occurs, as illustrated in Fig. 6. Specifically, in this application, the lateral misalignment will occur in the transmitting coil, which leads to a weakening of the magnetic coupling between the two coils. The consequences are reflected in the value of the mutual inductance, which will be evident in a considerable reduction in the value of the mutual inductance. Consequently, its value is determined based on Eq. (12) [17].

(12) $$M=\frac{\mu_{\circ }}{4\pi }\iint_{2\pi N}\frac{j\;d\theta_{1}d\theta_{2}}{\sqrt{R_{a}{}^{2}+R_{b}{}^{2}+h^{2}+x^{2}+-2j+2xR_{a}\cos\theta_{1}-2xR_{b}\cos\theta_{2}}}$$

The equations indicate that the primary parameters influencing M are the number of coil turns N, the inner and outer diameters, and the inter-coil spacing. Consequently, by regulating these factors, the value of M is managed.

### Multilayer Tx coil technology

**Fig. 7:**
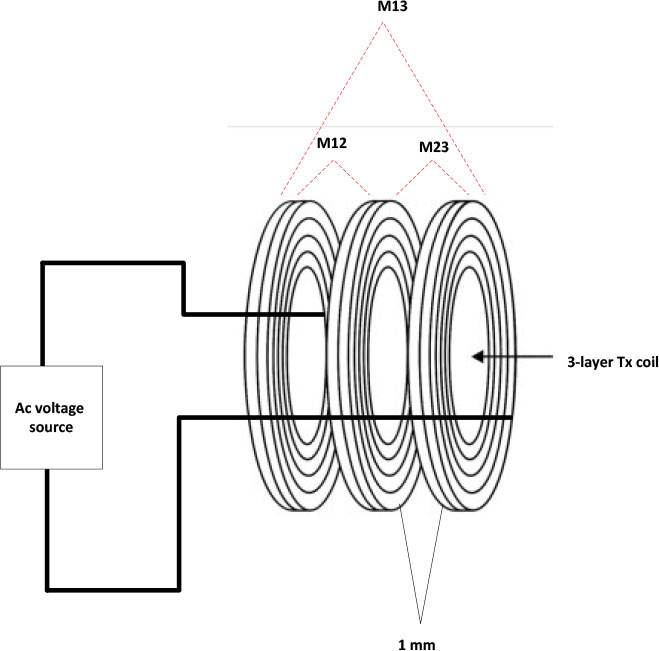
Three-layer Tx coil.

The WPT system's efficiency is correlated to the coils' self-inductance, which increases as the coil's diameter, number of turns, and number of layers increase [18]. The referencing coil is the transmitting coil, situated externally to the body, as the receiving coil is internal and its dimensions cannot be increased. Therefore, this work utilizes a multilayer coil approach, incorporating three layers of an identical-sized Tx coil, as shown in Fig. 7. The coil is constructed with identical wires to form these three layers, resulting in coils arranged in series. The self-inductance of the transmitting coil Tx is determined using Eq. (13) [18].

(13) $$L_{\mathrm{eq}}=L_{1}+L_{2}+L_{3}+2\left(M_{12}+M_{13}+M_{23}\right)$$

where *L*_1_, *L*_2_ *and L*_3_ represent the self-inductance of the first, second, and third layers of the Tx coil, and *M_1_, M_2_ and M_3_* represent the mutual inductance between layers, calculated according to Eq. (11).

### Designing a drive system

The transmission power varies with the changing distance between the transmitter and receiver in the WPT system. This variance is attributable to the circuit's input impedance (*Z_in_*), which varies with M variations. As the distance between the transmitting and receiving coils increased, mutual inductance (M) decreased, resulting in a reduction in input impedance (*Z_in_*) according to Eq. (14) [20]. To assess the efficacy of the WPT system, the transmitted power must remain constant.

(14) $$Z_{\mathrm{in}}=Z_{1}+\frac{w^{2}M^{2}}{Z_{1}+Z_{L}}$$

Power electronic converters manage voltage and ensure consistent power production. Therefore, the system comprises a non-inverting Buck-Boost Converter and an inverter. The power is controlled manually by adjusting the converter's duty cycle.

**Fig. 8:**
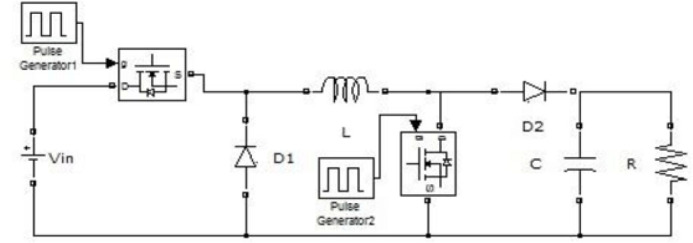
Non-inverting Buck-Boost converter.

The buck-boost converter has been constructed according to [19]. As shown in Fig. 8, this converter operates in a three-switching mode that depends on the duty cycle value (D). When D equals 0.5, the output voltage matches the input; when D surpasses 0.5, the converter functions as a boost converter. When D is less than 0.5, it functions as a buck converter. The output voltage is defined by Equation (15) [19].

(15) $$V_{o}=V_{\mathrm{in}}\frac{D}{1-D}$$

The minimum and maximum values of the inductor and capacitor for both the buck and boost converters were computed independently by the following equations [21,30]:

a) For buck converter:
  (16) $$L_{\min}=\frac{\left(1-D\right)R}{2f_{s}}$$
  (17) $$C_{\min}=\frac{1-D}{8L\left(\frac{\Delta V_{\mathrm{out}}}{V_{\mathrm{out}}}\right)f_{s^{2}}}$$
  where:
  *L_min_*: represents the minimum inductance required for the continuous current mode.

$\left(\frac{\Delta V_{\mathrm{out}}}{V_{\mathrm{out}}}\right)$ : represents the ripple as a function of output voltage.
  *f_s_*: the switching frequency.
  R is the load resistance, and its value is determined based on the input voltage and current of the WPT circuit when operating at a separation distance of 80 mm, ensuring an efficient converter design.
b) For the boost converter
  (18) $$L_{\min}=\frac{D{\left(1-D\right)}^{2}}{2f_{s}}$$
  (19) $$C_{\min}=\frac{D}{R\left(\frac{\Delta V_{\mathrm{out}}}{V_{\mathrm{out}}}\right)f_{s}}$$

The minimum values of L and C for both the buck and boost converters were calculated for D = 0.1 and D = 0.9. The inductance is 2 × 10^−3^ H, the capacitance is 10 × 10^−6^ F, and the resistance is 230 Ω, which verifies optimal performance and efficiency.

The H-bridge inverter was used in conjunction with a low-pass filter to eliminate the harmonics associated with the inverter output. The low-pass filter was designed according to Eq. (20), the resonant frequency equation, which was considered equal to the fundamental frequency of 6.78 MHz.

(20) $$f_{r}=\frac{1}{2\pi \sqrt{LC}}$$

### Human tissues and their influence on electromagnetic waves

The medium for transferring electromagnetic waves between the transmitter and receiver coils consists of a vacuum and human body tissues. As electromagnetic waves penetrate, a portion of energy is absorbed by the tissues, leading to problems in the propagation of electromagnetic waves and diminishing the electric field. This results in losses that directly impact the magnetic linkage between the two coils [22]. This occurs due to tissues' electrical and magnetic characteristics, including permittivity ɛ, conductivity σ, and permeability μ; hence, in most academic studies, tissues are modeled as a network of R-C impedances. The tissues of the human body that transmit electromagnetic waves are classified as lossy media, characterized by two parameters: the propagation constant β and the attenuation constant α. [23] provides a detailed study of how electromagnetic waves travel through the human body using Maxwell's equations to explain electromagnetic effects, sets up the equations for alpha and beta, and calculates how these two factors affect the electric and magnetic fields of the electromagnetic wave as shown in Eq. (21–22) [23].

(21) $$\alpha =\frac{w\sqrt{\mu \varepsilon }}{2}\sqrt{\sqrt{[1+{\left(\frac{\sigma }{w\varepsilon }\right)}^{2}}-1}(\text{NP}/\text{m})$$

(22) $$\beta =\frac{w\sqrt{\mu \varepsilon }}{2}\sqrt{\sqrt{[1+{\left(\frac{\sigma }{w\varepsilon }\right)}^{2}}+1}(\text{rad}/\text{m})$$

where σ, ɛ, and μ denote the dielectric properties of tissue, which are conductivity, permittivity, and permeability, respectively [31].

The transmitted electrical and magnetic field into the human tissues is given in the form [23]:

(23) $$E\left(z,t\right)=E_{\circ }e^{-\alpha z}\cos\left(\mathrm{wt}-\beta z\right)$$

(24) $$H\left(z,t\right)=H_{\circ }e^{-\alpha z}\cos\left(\mathrm{wt}-\beta z\right)$$

where w and z represent the angular frequency and tissue thickness, respectively. The magnitude of the electric and magnetic fields is affected by an exponential attenuation factor that depends on the thickness of the tissue and its dielectric properties. The dielectric properties of tissue represented by σ and ɛ at a frequency of 6.78 MHz were adopted by the IT'IS database based on Gabriel's study [24].

At the same time, μ was considered equal to the permeability of the vacuum because tissues are classified as non-magnetic materials [27, 28]. Individual body size and other factors, such as genetics, influence the tissue's variable thickness. The thickness of the epidermis varies between 0.06 and 0.1 mm, and the thickness of the dermis is 0.8 and 1.2 mm [23]. A study on cesarean sections, including 545 women, revealed that the muscle thickness in the upper left quadrant of the abdomen varied between 7.67 and 11.79 mm. In contrast, the fat layer thickness ranged between 15.47 and 31.31 mm [25]. The data also indicate that the thickness of the rib cage bone varied between 0.7 and 1.9 mm [26]. This study depended on the maximum thickness limits of these tissues to facilitate the propagation of electromagnetic waves towards the secondary coil integrated with the LVAD.

### Effect of tissues on the magnitude of mutual inductance

Eqs(23,24) represent the impact of tissues on the magnitude of mutual inductance, and its value is computed according to the following mathematical calculation. The magnetic flux density is:

(25) $$B=\mu_{\circ }\mu_{r}H$$

where, *μ_r_* is the permeability of the tissue. By substituting Eq.(24) in Eq.(25)

(26) $$B=B_{\circ }e^{-\alpha z}e^{-j\beta z}e^{\mathrm{jwt}}$$

The following equation gives the amount of magnetic flux from one coil that penetrates another coil:

(27) ∅=∫B dA

A denotes the cross-sectional area of the coil in *m*^2^. By substituting Eq.(23) in Eq.(24)

(28) $$\emptyset =B_{\circ }Ae^{-\alpha z}e^{-j\beta z}e^{\mathrm{jwt}}$$

∅_*mag*_ = *B*_○_ *Ae*^−*αZ*^, which is the magnitude of the magnetic flux and the amount of attenuation that will occur to it by an exponential factor. The magnitude of the mutual inductance depends directly on the magnitude of ∅.

(29) $$M_{\circ }=\frac{N_{2}\emptyset_{\circ }}{I_{1}}$$

where, ∅_○_ and *M*_○_ are the magnitude of flux and mutual inductance for vaccum.

(30) $$M_{\mathrm{mag}\_\mathrm{aten}}=\frac{N_{2}\emptyset_{\mathrm{mag}}}{I_{1}}$$

By substituting the equation ∅*_mag_* in Eq.(30)

(31) $$M_{\mathrm{mag}\_\mathrm{aten}}=\frac{N_{2}\emptyset_{\circ }e^{\_\alpha z}}{I_{1}}$$

(32) $$M_{\mathrm{mag}\_\mathrm{aten}}=M_{\circ }e^{-\alpha z}$$

### Efficiency calculation

Mutual inductance M is directly related to the coupling factor K, as expressed by Eq (33):

(33) $$K=\frac{M}{\sqrt{L_{1}L_{2}}}$$

The system's efficiency is assessed both theoretically and through simulation. A theoretical approach dependent on the quality factor of the coils Q and the coupling factor K, is based on Eq. (34) [22]. This method of efficiency calculation was employed to evaluate the coil design, assuming that the WPT system operates under ideal conditions, free of magnetic field dispersion or losses.

(34) $$\eta_{th}=\frac{K^{2}Q_{1}Q_{2}}{1+K^{2}Q_{1}Q_{2}+\frac{Q_{2}}{Q_{L}}}\times \frac{1}{1+\frac{L}{Q_{2}}}$$

(35) $$Q_{1}=\frac{WL_{1}}{R_{1}},\;\;Q_{2}=\frac{WL_{2}}{R_{2}}\;\;\mathrm{and}\;\;Q_{L}=\frac{R_{L}}{WL_{2}}$$

The system calculation efficiency from the simulation relies upon Eq. (36).

(36) $$\eta_{\mathrm{simu}}=\frac{P_{\mathrm{out}}}{P_{\mathrm{in}}}\times 100\%$$

### Research methodology flowchart

This section illustrates a flowchart outlining the procedures for designing the proposed system in this article.

### Simulink model

The Simulink model presented in this study comprises six main components: a 14 V DC power source, a Buck-Boost converter, an H-bridge inverter, a low-pass filter, the wireless power transfer (WPT) system, and a resistive load R, as illustrated in Fig. 11.

**Fig. 9:**
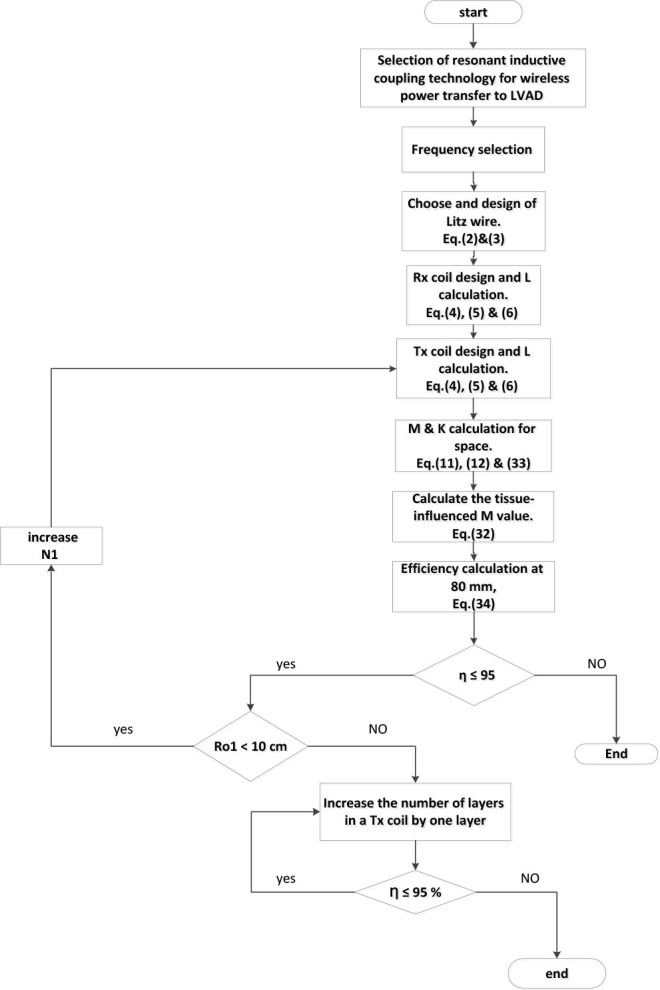
Coil design flowchart.

**Fig. 10:**
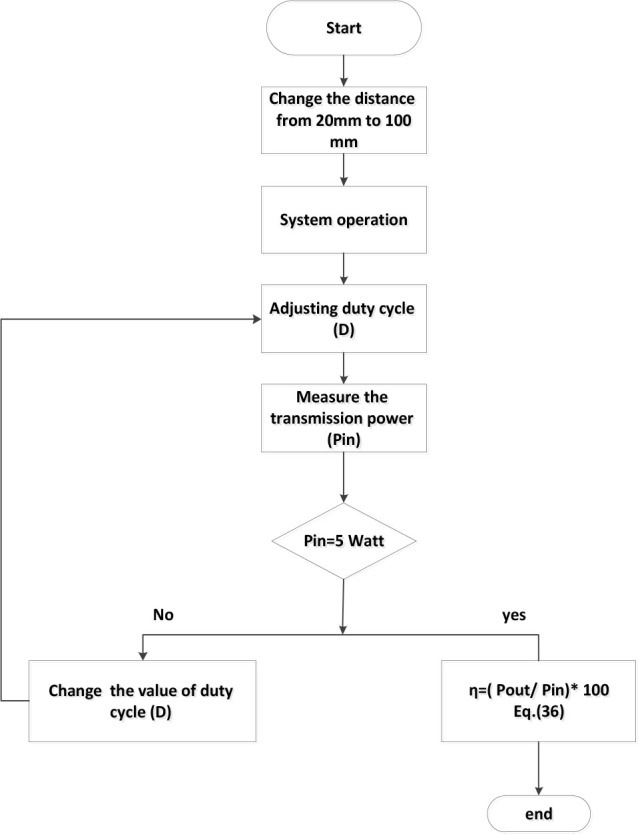
Transmission circuit power stabilization flowchart.

**Fig. 11:**
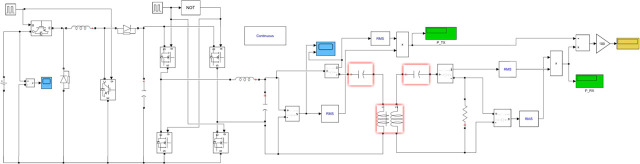
Simulink model of the proposed system.

### Ethical approval

The conducted research is not related to either human or animal use.

## Results and discussions

The proposed system was evaluated under two scenarios.

### First scenario: Central alignment

#### A: Single-layer Tx coil

**Fig. 12:**
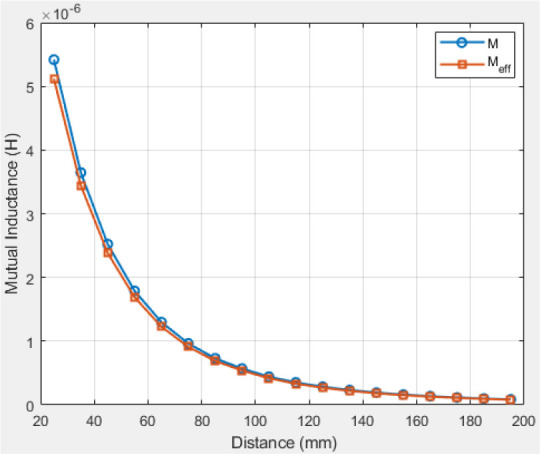
Mutual inductance at a single-layer Tx coil.

**Fig. 13:**
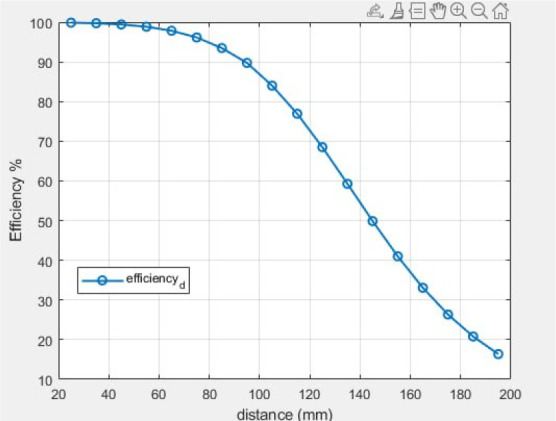
Theoretical efficiency of the WPT system at a single-layer Tx coil.

Fig. 12 demonstrates that mutual inductance diminishes with increasing distance for both vacuum (M) and tissue attenuation (Meff). The theoretical efficiency of the suggested system is illustrated in Fig. 13. The efficiency of the transmission system was evaluated using Eq.(31). Efficiency diminishes with increasing distance between the transmitter and receiver. The theoretical effectiveness of the WPT system was 98% at 60 mm and 69% at 140 mm.

#### B: Multilayer Tx coil

**Fig. 14:**
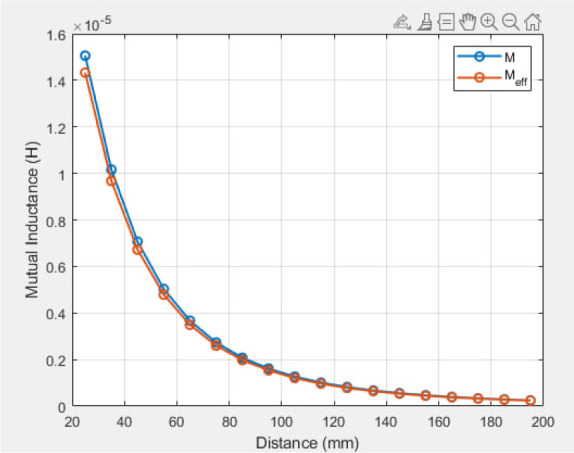
Mutual inductance at a multilayer Tx coil.

**Fig. 15:**
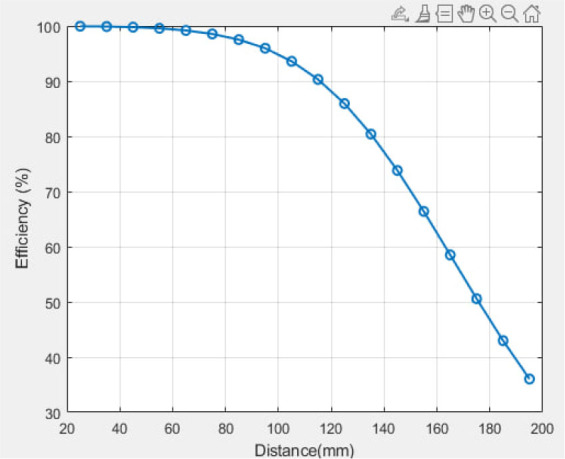
Theoretical efficiency of the WPT system at multilayer Tx coil.

Fig. 14 illustrates the mutual inductance as a function of distance following the utilization of a three-layer Tx coil. The mutual inductance increased by adding Tx layers, as shown in Fig. 12. The theoretically computed distance efficiency of the WPT system, as depicted in Fig. 15, increases with the separation distance, resulting in an 87% efficiency at 140 mm.

### Second scenario: lateral misalignment

The lateral alignment of the coils was modified in relation to the receiving coil diameter, which measured 0,1/4, 1/2, and 1/3 times the transmitting coil diameter of 70 mm, at a separation distance of 50 mm.

The mutual inductance value was computed for each x value, as depicted in Fig. 17, indicating that M decreases with increasing x. This is due to the decreasing magnetic coupling strength between the two coils as their lateral misalignment increases. The simulated efficiency was also calculated at each value of x, as illustrated in Table 2. Three variables change during these scenarios: the distance between the coils, the duty cycle, and the efficiency. Fig. 18 illustrates the correlation among these three elements, indicating that efficiency is inversely related to distance and directly related to the duty cycle.

**Fig. 16:**
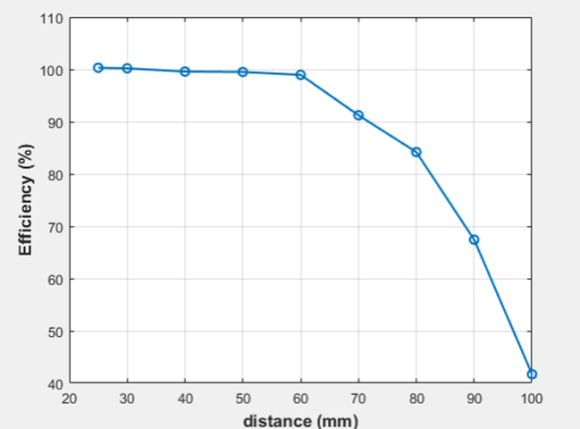
Simulated efficiecy of the proposed system.

**Fig. 17:**
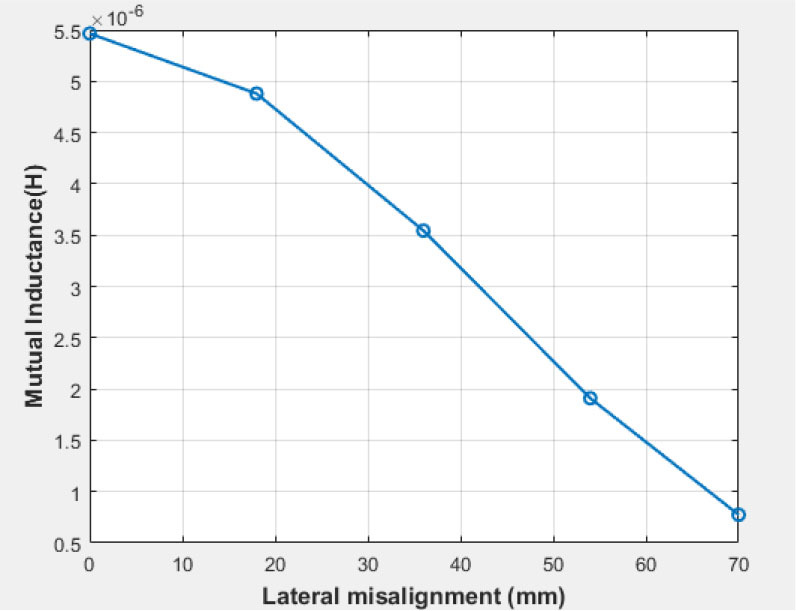
variation of mutual inductance with lateral alignment.

**Table 2:** Simulated efficiency at different values of x.

**X (mm)**	**Efficiency %**
0	99.5
18	99.0
36	95.9
54	76.0

**Fig. 18:**
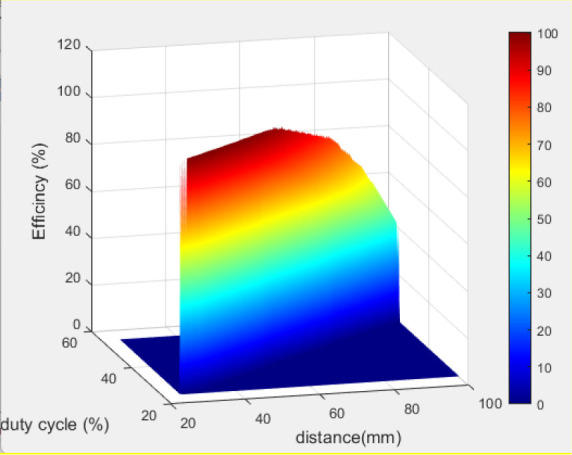
3D representation of the relationship between duty cycle, distance and efficiency.

## Conclusion

This study shows a wireless power transfer (WPT) system that uses inductive coupling to run an LVAD, focusing on being very efficient when the distance is between 20 mm and 100 mm. The system was optimized for a reference distance of 80 mm, where enhancements in coil quality and magnetic coupling improved transmission performance across larger gaps.

A mathematical model was developed to illustrate the influence of human tissue on magnetic coupling, and theoretical efficiency was assessed under various configurations. Results showed that with optimized coil design, efficiency remained high even at extended distances. Furthermore, the system's performance under lateral misalignment was analyzed, demonstrating a decline in efficiency as misalignment increased, underscoring the importance of precise coil alignment.

A drive system was also designed to stabilize the power in the transmitting circuit by controlling the duty cycle of a Buck-Boost converter manually. This research addresses a critical clinical limitation by offering a wireless solution that could eliminate driveline infections associated with LVADs. It also lays the groundwork for future research, particularly in assessing specific absorption rate (SAR) to ensure compliance with safety standards. The validated design in the MATLAB/Simulink environment supports future experimental implementation and clinical integration.
